# Level of structural integration in people with schizophrenia and schizoaffective disorders - applicability and associations with clinical parameters

**DOI:** 10.3389/fpsyt.2024.1388478

**Published:** 2024-06-06

**Authors:** Samuel Bayer, Anna-Lena Bröcker, Frauke Stuke, Sandra Just, Gianna Bertram, Imke Grimm, Eva Maaßen, Marielle Büttner, Andreas Heinz, Felix Bermpohl, Günter Lempa, Dorothea von Haebler, Christiane Montag

**Affiliations:** ^1^ Department of Psychiatry and Psychotherapy, Charité -Universitätsmedizin Berlin, Corporate Member of Freie Universität Berlin, Humboldt-Universität zu Berlin, and Berlin Institute of Health, Berlin, Germany; ^2^ International Psychoanalytic University Berlin, Berlin, Germany; ^3^ Psychotherapy Practice, Munich, Germany

**Keywords:** psychic structure, operationalized psychodynamic diagnosis, psychosocial functioning, severity of illness, psychosis, psychodynamic research, schizophrenia, schizoaffective disorder

## Abstract

**Introduction:**

The psychic structure of people with psychosis has been the subject of theoretical and qualitative considerations. However, it has not been sufficiently studied quantitatively. Therefore, the aim of this study was to explore the structural abilities of people diagnosed with schizophrenia and schizoaffective psychosis using the Levels of Structural Integration Axis of the Operationalized Psychodynamic Diagnosis System (OPD-2-LSIA). The study aimed to determine possible associations between the OPD-2-LSIA and central parameters of illness. Additionally, possible structural differences between people diagnosed with schizophrenia and schizoaffective psychosis were tested.

**Methods:**

This cross-sectional study included 129 outpatients with schizophrenia or schizoaffective disorders. Measures of structural integration, symptom load, severity of illness, cognition, and social functioning were obtained. Descriptive statistics were used to analyze the overall structural level and the structural dimensions. Correlation coefficients were computed to measure the associations between OPD-2-LSIA and variables regarding the severity of illness and psychosocial functioning. Regression models were used to measure the influence of illness-related variables on OPD-2-LSIA, and the influence of OPD-2-LSIA on psychosocial functioning. Participants diagnosed with schizophrenia and schizoaffective disorders were examined with regard to possible group differences.

**Results:**

The results of the OPD-2-LSIA showed that the overall structural level was between ‘moderate to low’ and ‘low level of structural integration’. Significant correlations were found between OPD-2-LSIA and psychotic symptoms (but not depressive symptoms), as well as between OPD-2-LSIA and psychosocial functioning. It was found that variables related to severity of illness had a significant impact on OPD-2-LSIA, with psychotic, but not depressive symptoms being significant predictors. OPD-2-LSIA was found to predict psychosocial functioning beyond symptoms and cognition. No significant differences were found between participants with schizophrenia and schizoaffective psychosis. There was also no correlation found between OPD-2-LSIA and depressive symptomatology (except for the subdimension Internal communication).

**Discussion:**

Contrary to theoretical assumptions, the results of the study show a heterogenous picture of the psychic structure of people with psychosis. The associations between OPD-2-LSIA and severity of illness, particularly psychotic symptomatology, as well as the influence of OPD-2-LSIA on psychosocial functioning, are discussed.

## 1 Introduction

The interest in psychotherapeutic approaches to treating psychotic disorders has steadily increased over the years. Besides pharmacotherapy, psychotherapy has become an integral part of the treatment recommended by national and international guidelines (1, 2). While the efficacy of cognitive behavioral therapy (CBT) and family interventions (i.a. 3–5) has been proven, a lack of quantitative research prevents a statement about the efficacy of psychodynamic psychotherapy (PDT) (i.a. 6, 7). However, there is a long history of PDT for people with psychosis with a clinical consensus that technical modifications are required (7–9). The assessment of inner psychic structural integration is considered an important component for the decision on the differential indication for PDT and outlines the framework for setting, treatment goals and specific interventions (10–12). The concept of structural integration has a long tradition in psychoanalytic theory and research (i.a. 13). Psychic structure defines the “vulnerability of the personality, the disposition to illness and the capacity to process internal conflicts and external experiences of stress” (14, p.199). The concept was developed further by Otto Kernberg, who postulated three relatively stable levels of personality structure depending on the maturity of mental functioning: neurotic, borderline and psychotic personality organization (15). Until now, research on psychic structure has been mainly relevant in psychodynamic and psychoanalytic discussions. However, it could acquire a broader relevance as the proximity between the Levels of Personality Functioning Scale (LPFS) for personality disorders in the Alternative DSM-5 Model for Personality Disorders (AMPD; 16) and OPD-2-LSIA has been demonstrated (17–19). From a psychodynamic perspective, the levels of personality functioning are not only of interest in the context of personality disorders (20). Rather, they determine the vulnerability to disorders and the ability to cope with interpersonal challenges and stress in general.

Empirical measures for the assessment of psychic structure are the Structured Interview of Personality Organization (STIPO; 21) or Wallerstein’s Scales of Psychological Capacities (SPC; 22). Another approach is the Axis IV of the multiaxial Operationalized Psychodynamic Diagnosis (OPD-2; 14), an instrument that aims at integrating different psychoanalytic strands of thought. The “Levels of Structural Integration Axis” of the OPD-2 (OPD-2-LSIA) has been widely used and proven good psychometric properties in numerous studies (18, 23–25). OPD-LSIA has been applied to various disorders such as somatoform disorders, unipolar mood and anxiety disorders, or to distinguish between patient groups with and without personality disorder (23, 26–29). Although the psychic structure of people with psychosis has been the subject of many psychodynamic considerations, it has not been sufficiently studied quantitatively. Based on theory, it is assumed that people with psychosis have the “lowest” or “weakest” level of structural integration, consequently a “psychotic” or “disintegrated” psychic structure (14, 15). There is only one small study which has used OPD-LSIA in people with schizophrenia (30). Additionally, there is a single case study in which the applicability and usefulness of OPD-2 for bipolar disorders was examined (31).

Psychic structure is described as being rather invariant (15, 32), independent of biological sex (33), age (33, 34), education (10) and current symptomatology (14). The results of Spitzer et al., who found “at best only slight” (33, p. 396) correlations between the OPD-LSIA (surveyed using OPD-1; 35) and subjective symptom-related distress (using the Symptom Checklist-90-R; SCL-90-R) in mixed samples, support this notion of a stable, symptom-independent structure. By contrast, other studies found significant associations between psychic structure and symptom-related distress (26, 36, 37). Studies using the OPD-Structure Questionnaire (OPD-SQS; 38) have expanded the psychic structure research in recent years and have shown significant associations between OPD-SQS and symptom severity, symptomatic change, etc. (e.g. 39, 40). In psychoses, however, associations between psychic structure and severity of illness or specific symptomatology have not been investigated. It is assumed that exposure to early traumatization and deprivation hinder structural integration (41) as well as represent a risk-factor for psychotic disorders (42).

Besides symptomatology and illness severity, psychosocial functioning is a central outcome parameter in the treatment of mental disorders and particularly relevant in people with psychosis (43). Despite theoretical links between behavior in the sense of psychosocial adaptation and psychic structure (14, 32), studies on psychosocial functioning are underrepresented in OPD-2-LSIA research and have not been conducted specifically within the field of psychosis.

Several studies have demonstrated prognostic differences between schizoaffective and schizophrenic psychoses (44, 45). With regard to structural abilities, however, there have been no studies to date that examined possible differences in these patient groups. Yet, in a mixed sample, an association was found between a higher structural level and a better prognosis (7-year follow-up; 46).

Traditionally, the presence of affective symptoms, especially, is considered an indicator for a more favorable course of the illness (47–49). However, a possible correlation between depressive symptom load and structural abilities in people with psychosis has not been tested yet.

With regard to the state of research, the aim of this study was to explore the structural abilities of persons diagnosed with schizophrenia and schizoaffective psychosis. The study examined structural abilities on a descriptive level. Additionally, associations between OPD-2-LSIA and parameters of severity of illness and between OPD-2-LSIA and parameters of psychosocial functioning were investigated. Furthermore, the study compared the structural abilities of people diagnosed with schizophrenia and schizoaffective psychosis. This led to the following hypotheses:

1a) There are significant negative correlations between structural abilities (OPD-2-LSIA) and severity of illness as well as psychotic symptom load. 1b) OPD-2-LSIA is predicted by state-related (current symptomatology, CGI-S, inpatient treatment days in the last two years) as well as long-term (duration of illness, lifetime hospital stays, WHO-DDD, childhood maltreatment) indicators of illness severity, but not by sociodemographic (age, biological sex) and cognitive (verbal memory and learning, verbal IQ) factors.
2a) A significant positive correlation between parameters of psychosocial functioning (Mini-ICF-APP, Modified Vocational Status Index, Modified Location Code Index) and structural abilities (OPD-2-LSIA) can be assumed. 2b) Psychosocial functioning (Mini-ICF-APP) is predicted by OPD-2-LSIA.
3a) People with schizophrenia show less favorable OPD-2-LSIA values than people with schizoaffective psychosis. 3b) There is a significant positive correlation between structural abilities (OPD-2-LSIA) and depressive symptom load.

## 2 Materials and methods

### 2.1 Participants and procedure

129 outpatients aged between 19 and 63 years gave written informed consent and were included in the baseline sample of the study “Modified Psychodynamic Psychotherapy for Patients with Schizophrenia – a Randomized-Controlled Efficacy Study” (MPP-S; ClinicalTrials.gov-ID: NCT02576613). The study was approved by the ethics committee of the Charité Universitätsmedizin Berlin. Subsamples have already been published with other research questions (50–53). Participants were *n* = 74 male and *n* = 55 female outpatients meeting the DSM-IV-TR criteria of schizophrenia or schizoaffective disorder (Diagnostic and Statistical Manual of Mental Disorders; 54). Diagnoses were confirmed by an experienced psychiatrist using the structured Interview for DSM-IV (SCID-I; 55). Participants in all phases of the illness were included in the study. No subdivision was made in terms of acuity, or in terms of psychotic or thymic phases. However, all patients were at least partially remitted and stabilized, so no inpatient admission was necessary. The mean World Health Organization defined daily dose of antipsychotics (WHO-DDD; 56) was M = 1.19 (± 1.30; 0–12.14). Additionally, *n*=9 participants were treated with mood stabilizers, and *n*=27 participants with antidepressants. Criteria for exclusion were any other DSM-IV axis-I disorder, organic brain disease or somatic illnesses affecting cerebral function, acute suicidality, indication for a primary addiction-specific treatment, or acute endangerment of others. Sociodemographic data and characteristics of illness are presented in **Table 1**.

**Table 1:** Characteristics of participants.

Variable	N	Value
**Biological sex**	129	
Male (%)	74	57.4 %
Female (%)	55	42.6 %
**Age** (± SD in years; R)	129	M = 37.86 (± 11.32; 19-63)
**Diagnosis**	129	
Schizophrenia (%) Schizoaffective disorder (%)	96 33	74.4 % 25.6 %
**WHO-DDD** (± SD; R)	129	M = 1.19 (± 1.30; 0–12.14)
**Duration of illness** (± SD in years; R)	129	M = 13.10 (± 9.78; 0-46)
**Lifetime psychiatric hospital stays** (± SD; R)	128	M = 4.77 (± 4.79; 0-28)
**Inpatient treatment days in the last two years** (± SD; R)	125	M = 43.96 (± 62.78; 0-330)
**Years of education** (± SD in years; R)	128	M = 15.23 (± 3.19; 9-24)
**Verbal IQ** (± SD; R)	127	M = 105.36 (± 12.02; 72-129)
**AVLT mean value** (± SD; R)	125	M = 9.00 (± 2.52; 2.2–14)
**Modified Vocational Status Index (MVSI)**	129	
Employed full-time at expected level (%) Employed full-time below expected level (%) Employed part-time (%) Vocational (re-)training (%) Retired with an additional income, volunteer work or active housewife/ -husband (%) Employed in a sheltered workshop (%) Occupational therapy, day care center (%) Long-term unemployed but employable (%) Retired or unable to work (%)	23 6 11 7 8 15 4 33 22	17.8 % 4.7 % 8.5 % 5.4 % 6.2 % 11.6 % 3.1 % 25.6 % 17.1 %
**Modified Location Code Index (MLCI)**	129	
Living in a relationship (as a parent, in a partnership, with friends or relatives) (%) In an autonomous household alone, with peers or shared apartment (%) With the nuclear family (%) In an autonomous household with minimal supervision (%) In a therapeutic residential community (%) In a household with intensive supervision (%) In transitional housing with intensive care (%) In a nursing home (%) In a permanent homeless shelter (%) Homeless (%)	18 51 7 36 11 2 2 0 2 0	14 % 39.5 % 5.4 % 27.9 % 8.5 % 1.6 % 1.6 % 0 % 1.6 % 0 %
**PANSS total** (± SD; R) PANSS cognition (± SD; R) PANSS depression (± SD; R) PANSS excitement (± SD; R) PANSS negative (± SD; R) PANSS positive (± SD; R)	129 129 129 129 129 129	M = 58.93 (± 15.05; 31-106) M = 16.08 (± 5.19; 9-36) M = 11.00 (± 3.88; 5-24) M = 5.55 (± 1,84; 4-13) M = 15.28 (± 6.10; 7–34) M = 11.02 (± 4.92; 5–24)
**CDSS total** (± SD; R)	129	M = 5.93 (± 5.33; 0-24)
**SANS total** (± SD; R) SANS affective flattening (± SD; R) SANS alogia (± SD; R) SANS apathy (± SD; R) SANS anhedonia (± SD; R) SANS attention (± SD; R)	129 129 129 129 129 129	M = 25.30 (± 16.90; 0-96) M = 5.57 (± 6.93; 0-53) M = 2.77 (± 4.05; 0-16) M = 6.06 (± 3.90; 0-15) M = 9.50 (± 5.93; 0-22) M = 1.40 (± 2.43; 0-9)
**SAPS total** (± SD; R) SAPS hallucination (± SD; R) SAPS delusion (± SD; R) SAPS bizarre behavior (± SD; R) SAPS positive formal thought disorder (± SD; R) SAPS inappropriate affect (± SD, R)	129 129 129 129 129 129	M = 18.16 (± 16.80; 0-83) M = 4.54 (± 6.25; 0-25) M = 7.48 (± 7.46; 0-32) M = 0.89 (± 1.86; 0-10) M = 4.96 (± 6.04; 0-27) M = 0.28 (± 0.74; 0-3)
**GAF** (± SD; R)	129	M = 58.44 (± 12.68; 30-85)
**Mini-ICF-APP total** (± SD; R)	129	M = 18.04 (± 8.49; 1-37)

Frequencies are presented in percentage; N, number; M, mean; SD, standard deviation; R, range; WHO-DDD, World Health Organization defined daily dose; Verbal IQ, Wortschatztest WST; AVLT, Auditory Verbal Learning Test; PANSS, Positive and negative Syndrome Scale, five-factor structure (57); CDSS, Calgary Depression Scale for Schizophrenia; SANS, Scale for the Assessment of Negative Symptoms; SAPS, Scale for the Assessment of Positive Symptoms; GAF, Global Assessment of Functioning; Mini-ICF-APP, Mini-ICF-Rating for Limitations of Activities and Participation in Psychological disorder.

### 2.2 Instruments

#### 2.2.1 Clinical interview

A semi-structured interview lasting between 45 and 90 minutes and based on the recommendations given by the OPD-2 task force (14) was conducted by two members of a pool of trained psychologists and psychiatrists. Assessments were made based on a consensus rating. For a detailed description of the interview, see Bröcker et al. (50) or Stuke et al. (53).

#### 2.2.2 Operationalized psychodynamic diagnosis

OPD-2 (14) is a multiaxial psychodynamic diagnostics system with five axes: Experience of illness and prerequisites for treatment (axis I), Interpersonal Relations (axis II), Conflict (axis III), Levels of Structural Integration Axis (OPD-2-LSIA; axis IV) and Mental and Psychosomatic Disorders (axis V). OPD-2-LSIA consists of 24 items which are differentiated across four dimensions, each with relation to the self and to (external and internal) objects, i.e. relationships with others and internal representation of significant others (14). This leads to eight (4 x 2) structural dimensions, each consisting of three structural items, which are assessed by four classificatory levels of structural integration. Moreover, there are three interim classifications, that can be used if the structural ability exhibits parts of the two adjacent assessment dimensions. The assessment dimensions are assigned a seven-stage Likert scale. Higher ratings indicate lower structural capabilities (1 – High level of structural integration, 1.5, 2 – Moderate level of structural integration, 2.5, 3 – Low level of structural integration, 3.5, 4 – Disintegrated level of structural integration). The rating of the items leads to a profile of structural abilities for each individual structure dimension and a sum score indicating the overall structural level. OPD-2-LSIA is rated based on the previous two years in a person’s life (14). Several studies have demonstrated the reliability and validity of the OPD-LSIA, for an overview see Zimmermann et al. (18). The structural dimensions and items are presented in **Table 2**.

**Table 2:** OPD-2-LSIA Levels of structural integration .

OPD-2-LSIA	Subdimension/Items	N	Value (± SD; R)
**Cognitive abilities**	Self-perception 1.1 Self-reflection 1.2 Affect differentiation 1.3 Identity	129	M = 2.56 (± 0.63; 1.50-4.00)
	Object perception 1.4 Self-object differentiation 1.5 Whole object perception 1.6 Realistic object perception	129	M = 2.99 (± 0.59; 1.33-4.00)
**Capacities for regulation**	Self-regulation 2.1 Impulse control 2.2 Affect tolerance 2.3 Self-worth regulation	129	M = 2.77 (± 0.46; 2.00-4.00)
	Regulation of object-relationship 2.4 Protecting relationships 2.5 Balancing of interests 2.6 Anticipation	129	M = 2.75 (± 0.51; 1.67-4.00)
**Emotional abilities**	Internal communication 3.1 Experiencing affects 3.2 Use of fantasies 3.3 Bodily self	129	M = 2.74 (± 0.599; 1.00-4.00)
	Communication with the external world 3.4. Making contact 3.5 Communication of affect 3.6 Empathy	129	M = 2.60 (± 0.61; 1.33-4.00)
**Attachment capacities**	Internal objects 4.1 Internalization 4.2 Use of introjects 4.3 Variability of attachment patterns	129	M = 2.96 (± 0.53; 1.83-4.00)
	External objects 4.4 Ability to make attachments 4.5 Accepting help 4.6 Severing attachments	129	M = 3.07 (± 0.56; 1.67-4.00)
	Overall structural level	129	M = 2.80 (± 0.46; 1.75-3.90)

N, number; M, mean; SD, standard deviation; R, range.

OPD-2-LSIA values: 1 – High level of structural integration, 1.5 – High to moderate level of structural integration, 2 – Moderate level of structural integration, 2.5 – Moderate to low level of structural integration, 3 – Low level of structural integration, 3.5 – Low to disintegrated level of structure, 4 – Disintegrated level of structure.

#### 2.2.3 Symptomatology and severity of illness

To assess symptom, load a five factor model of the Positive and Negative Syndrome Scale (PANSS; 57) was used, including positive and negative symptoms, cognition, depression/anxiety and excitement/hostility (58). Additionally, the Scale for the Assessment of Positive Symptoms (SAPS; 59), the Scale for the Assessment of Negative Symptoms (SANS; 60) and the Calgary Depression Scale for Schizophrenia (CDSS-G; 61) were completed. All instruments proved good psychometric properties (e.g. 61–64). Data on psychopathology is given in **Table 1**.

Next to the current symptomatology the severity of illness was assessed with different variables: Clinical Global Impression Severity scale (CGI-S; 65) and psychiatric inpatient treatment days in the last two years as state-related indicators. Duration of illness, lifetime psychiatric hospital stays, childhood maltreatment and the World Health Organization defined daily dose of antipsychotics (WHO-DDD; 56) as long-term indicators. WHO-DDD was estimated a long-term indicator as participants were stabilized under long-term medication. To assess childhood maltreatment the Childhood Trauma Screener (CTS; 66) was used.

#### 2.2.4 Psychosocial functioning

Psychosocial functioning was measured with the Mini-ICF-Rating for Limitations of Activities and Participation in Psychological disorder (Mini-ICF-APP; 67). The Mini-ICF-APP was built in reference to the International Classification of Function, Disability and Health (68) and consists of thirteen dimensions of capacity, each rated on a five-point Likert-scale from ‘no impairment’ to ‘total disability’, higher values indicating more severe impairment (69). The Mini-ICF-APP is a validated, reliable and efficient instrument for measuring impairments in persons with mental disorders (69, 70).

Additionally, the Modified Vocational Status Index (MVSI) (71) and the Modified Location Code Index (MLCI) (71) were used as measurements for psychosocial functioning regarding occupational and residential status. The indices were adjusted for the German care system. The MVSI is a nine-point scale to assess the level of occupational functioning, descending from 1 (indicating employed full-time at expected level) to 9 (retired or unable to work). The MLCI is a ten-point scale to assess the living situation, descending from 1 (living as a parent with children, in a partnership, with friends or relatives) to 10 (homeless, on the streets). The anchor points are provided in the **Supplementary Material**.

#### 2.2.5 Cognitive functioning

Verbal memory and learning were assessed with the German version of the Auditory Verbal Learning Test (AVLT; 72). The level of verbal IQ was determined by using a multiple-choice vocabulary test (Wortschatztest WST; 73).

### 2.3 Statistical analysis

A principal component analysis (PCA) was conducted to reduce data of the four symptom scales (PANSS, SAPS, SANS, CDSS), to avoid alpha-error inflation, and to bundle the existing subfactors in the symptom assessment scales into one model for further calculations. PCA identified four factors: ‘Factor 1 Cognition/Negative symptoms’, ‘Factor 2 Delusion/Hallucinations’, ‘Factor 3 Depression’ and ‘Factor 4 Excitement/Disorganization’. A more detailed description of the factor analysis can be found in Bröcker et al. (50).

Descriptive statistical analysis was applied to the overall structural level and individual structural dimensions. Spearman correlation coefficients were computed between OPD-2-LSIA and variables regarding the severity of illness (specific psychotic symptom load, duration of illness, CGI-S, WHO-DDD, inpatient treatment days in the last two years, lifetime hospital stays, childhood maltreatment) as well as between OPD-2-LSIA and psychosocial functioning (Mini-ICF-APP, MVSI and MLCI).

A multiple linear regression was conducted to measure the individual contributions of illness-related variables on OPD-2-LSIA. Next to age, biological sex and cognitive functioning (AVLT and WST), the current symptomatology, and the variables regarding the severity of illness were used as independent variables. All variables were entered simultaneously (enter-method).

To analyze the influence of OPD-2-LSIA on psychosocial functioning (Mini-ICF-APP), a hierarchical multiple linear regression was calculated. Socio-demographic data, cognitive functioning and the current symptom load were used as covariates. Socio-demographic data (age, biological sex) was entered in the first block, education specific and cognitive functioning (AVLT, WST) in the second block. Symptom load (Factor 1 Cognition/Negative symptoms, Factor 2 Delusion/Hallucinations, Factor 3 Depression, Factor 4 Excitement/Disorganization) was entered in the third block, OPD-2-LSIA in the fourth block.

Two-sided t-tests for independent samples were used to determine group differences in OPD-2-LSIA ratings between participants diagnosed with schizophrenia and schizoaffective disorder. Spearman correlation analyses were performed to assess correlations between OPD-2-LSIA and depression specific symptomatology.

The different statistical test methods (linear regressions, t-test, Spearman correlations, ANOVA) were assessed in terms of their underlying assumptions such as lack of multicollinearity, normality, linearity, outliers (casewise diagnostics) and homoscedasticity. P **≤** 0.05 was assumed as the level of significance for all calculations. To control type I error in multiple comparisons, Bonferroni-Holm corrections were used. The statistical analyses were conducted using SPSS-23 (74).

## 3 Results

Mean OPD-2-LSIA values for the total scale (overall structural level), as well as for the individual structural dimensions, were between ‘moderate to low level of structural integration’ and ‘low level of structural integration’ (*M*=2.80, *R*= 1.75 - 3.90, *SD*=0.46). Least structural limitations were found in the dimensions Self-perception and Communication with the external world, while the greatest structural limitations were found in the dimensions Object perception, Internal objects and External objects. See **Table 2** for detail.

### 3.1 Hypotheses 1a and 1b

Regarding hypothesis 1a), significant correlations were found between the overall structural level and the CGI-S (*r_s_* =0.586, *p* < 0.001), the inpatient treatment days in the last two years (*r_s_* =0.176 *p*=0.049) and the lifetime hospital stays (*r_s_* =0.252, *p*=0.004). No correlations were found between the overall structural level and WHO-DDD (*r_s_* =0.035, *p*=0.697), childhood maltreatment (*r_s_* =0.162, *p*=0.111), as well as duration of illness (*r_s_* =0.069, *p*=0.436). Regarding the current symptom load a significant correlation was found between the overall structural level and the specific psychotic symptom factors: Factor 1 Cognition/Negative symptoms, Factor 2 Delusion/hallucinations and Factor 4 Excitement/disorganization. There were relevant associations between the three factors and all structural dimensions. After Bonferroni-Holm correction, the correlation coefficients between Factor 4 Excitement/disorganization and the dimensions Internal communication and (Attachment to) External objects did not remain significant. All results are presented in detail in **Table 3**.

**Table 3:** Correlation coefficients between symptom factors and OPD-2-LSIA dimensions.

OPD-2-LSIA Dimensions		Factor 1 Cognition/Negative symptoms	Factor 2 Delusion/Hallucinations	Factor 3 Depression	Factor 4 Excitement/disorganization
**Self-perception**	r_s_	.314^**^	.310^**^	-.047	.298^**^
	p	**<.001**	**<.001**	.597	**<.001**
**Object perception**	r_s_	.324^**^	.441^**^	.060	.248^**^
	p	**<.001**	**<.001**	.502	**.005**
**Self-regulation**	r_s_	.207^*^	.338^**^	.138	.333^**^
	p	**.018**	**<.001**	.118	**<.001**
**Regulation of object-relationship**	r_s_	.343^**^	.270^**^	.051	.286^**^
	p	**<.001**	**.002**	.567	**.001**
**Internal communication**	r_s_	.426^**^	.408^**^	.250^**^	.193^*^
	p	**<.001**	**<.001**	**.004**	**.029**
**Communication with the external world**	r_s_	.450^**^	.208^*^	-.062	.308^**^
	p	**<.001**	**.018**	.487	**<.001**
**Internal objects**	r_s_	.255^**^	.443^**^	.036	.299^**^
	p	**.003**	**<.001**	.688	**<.001**
**External objects**	r_s_	.301^**^	.327^**^	.139	.176^*^
	p	**<.001**	**<.001**	.117	**.046**
**Overall structural level**	r_s_	.424^**^	.420^**^	.079	.323^**^
	P	**<.001**	**<.001**	.373	**<.001**

Spearman’s rank correlations; OPD-2-LSIA, Levels of Structural Integration Axis of the Operationalized Psychodynamic Diagnosis System.

*p <.05; **p <.01; bold = significance maintained after Bonferroni-Holm method.

With regard to hypothesis 1b), a multiple regression analysis was conducted (see **Table 4**), indicating that the severity of illness variables may have a significant impact on the overall structural level (F(14, 76) = 9.112, p < 0.001), explaining 53.3% of the variance (adjusted R^2^ = 0.558). The psychotic symptom factors (Factor 1 Cognition/Negative symptoms, Factor 2 Delusion/Hallucinations, Factor 4 Excitement/disorganization) are significant predictors of the overall structural level.

**Table 4:** Impact of cognitive and illness-related variables on OPD-2-LSIA overall structural level.

Predictor	OPD-2-LSIA Overall structural level			
	B	SE B	ß	p
Constant	2.325	.391		<.001
**Age**	-.009	.005	-.224	.088
**Biological sex**	-.013	.075	-.015	.864
**Verbal IQ**	.003	.003	.074	.362
**AVLT mean**	.026	.015	.144	.097
**Duration of illness**	.012	.006	.255	.053
**CGI-S**	.025	.048	.061	.593
**Lifetime psychiatric hospital stays**	.008	.007	.097	.218
**Inpatient treatment days in the last two years**	.001	.001	.100	.205
**WHO-DDD**	-.026	.040	-.054	.513
**Childhood maltreatment**	-.002	.011	-.017	.838
**Factor 1 Cognition/Negative symptoms**	.206	.048	.431	<.001
**Factor 2 Delusion/Hallucinations**	.192	.037	.436	<.001
**Factor 3 Depression**	.056	.035	.127	.109
**Factor 4 Excitement/disorganization**	.164	.033	.390	<.001

Multiple regression analysis; dependent variable: OPD-2-LSIA, Levels of Structural Integration Axis of the Operationalized Psychodynamic Diagnosis System. Covariates are: Age; Biological sex; Verbal IQ, Wortschatztest WST; AVLT mean, Auditory Verbal Learning Test, mean score of the five initial presentations; Duration of illness; CGI-S, Clinical global impressions Severity scale; Lifetime psychiatric hospital stays; Inpatient treatment days in the last two years; WHO-DDD, World Health Organization defined daily dose; Childhood maltreatment; Factor 1 Cognition/Negative symptoms; Factor 2 Delusion/Hallucinations; Factor 3 Depression; Factor 4 Excitement/disorganization.

### 3.2 Hypotheses 2a and 2b

Regarding hypothesis 2a), a significant correlation was found between the sum score of impairments of activity and participation (Mini ICF-AAP) and the overall structural level, as well as between the overall structural level and the occupational status (MVSI), and the residential status (MLCI). See **Table 5** for details.

**Table 5:** Correlation coefficients between OPD-2-LSIA overall structural level and the social functioning variables.

OPD-2-LSIA		Mini-ICF-APP	MVSI	MLCI
**Overall structural level**	r_s_	.619**	.238**	.274**
	P	<.001	.007	.002

Spearman’s rank correlations; OPD-2-LSIA, Levels of Structural Integration Axis of the Operationalized Psychodynamic; Mini-ICF-APP, Mini-ICF-Rating for Limitations of Activities and Participation in Psychological disorder; MVSI, Modified Vocational Status Index Diagnosis System; MLCI, Modified Location Code Index; **p <.01.

To test hypothesis 2b), a hierarchical multiple regression analysis was conducted (see **Table 6**). Sociodemographic data added in the first block did not prove to be a significant predictor. Cognitive variables were added in the second block (adjusted R² = 0.069, R² change = 0.066, F(2, 119) = 3.268, p= 0.014). Current symptom load (adjusted R² = 0.690, R² change = 0.611, F(4, 115) = 35.227, p<.001) added in the third block, as well as OPD-2-LSIA (adjusted R² = 0.745, R² change = 0.054, F(1, 114) = 40,981, p< 0.001), added in the fourth block, were significant predictors explaining significantly more variance. The last two blocks received the lowest Akaike information criterion scores (AIC; 75), indicating that these models were the best fitting models for the data on hand. In sum, OPD-2-LSIA remained a significant predictor of psychosocial functioning, after effects of sociodemographic data, cognitive variables and present symptom load were accounted for.

**Table 6:** Impact of symptomatology and OPD-2-LSIA overall structural level on Mini-ICF-APP.

Block		Mini-ICF-APP				
		B	SE B	ß	p	AIC
**Block 1**	Constant	19.393	2.581		<.001	
	**Age**	-.013	.067	-.017	.851	
	**Biological sex**	-2.921	1.515	-.176	.056	
						524.380
**Block 2**	Constant	25.123	6.664		<.001	
	**Age**	-.060	.072	-.082	.411	
	**Biological sex**	-1.965	1.524	-.118	.200	
	**Verbal IQ**	.037	.070	.052	.600	
	**AVLT mean**	-.910	.311	-.277	.004	
						519.562
**Block 3**	Constant	16.343	3.991		<.001	
	**Age**	-.001	.043	-.001	.980	
	**Biological sex**	-.655	.916	-.040	.476	
	**Verbal IQ**	.031	.041	.044	.453	
	**AVLT mean**	-.140	.188	-.043	.457	
	**Factor 1 Cognition/Negative symptoms**	3.822	.458	.456	<.001	
	**Factor 2 Delusion/Hallucinations**	3.015	.428	.365	<.001	
	**Factor 3 Depression**	5.253	.431	.626	<.001	
	**Factor 4 Excitement/disorganization**	1.395	.443	.162	.002	
						386.909
**Block 4**	Constant	-1.036	4.974		.835	
	**Age**	-.008	.039	-.011	.836	
	**Biological sex**	-.661	.830	-.040	.428	
	**Verbal IQ**	.029	.037	.041	.435	
	**AVLT mean**	-.186	.171	-.056	.280	
	**Factor 1 Cognition/Negative symptoms**	2.394	.501	.286	<.001	
	**Factor 2 Delusion/Hallucinations**	1.567	.481	.190	.001	
	**Factor 3 Depression**	4.849	.398	.577	<.001	
	**Factor 4 Excitement/disorganization**	.148	.470	.017	.753	
	**OPD-2-LSIA Overall structural level**	6.497	1.276	.355	<.001	
						363.498

Hierarchical regression analysis; dependent variable: Mini-ICF-APP, Mini-ICF-Rating for Limitations of Activities and Participation in Psychological disorder; covariats are: block 1: Age; Biological sex; Block 2: Verbal IQ, Wortschatztest WST; AVLT mean, Auditory Verbal Learning Test Auditory Verbal Learning Test, mean score of the five initial presentations; Block 3: Factor 1 Cognition/Negative symptoms; Factor 2 Delusion/Hallucinations; Factor 3 Depression; Factor 4 Excitement/disorganization; Block 4: OPD-2-LSIA Overall structural level, Levels of Structural Integration Axis of the Operationalized Psychodynamic Diagnosis System, overall structural level; AIC, Akaike information criterion.

### 3.3 Hypotheses 3a and 3b

Performing t-tests, there were no significant differences between participants with schizophrenia and schizoaffective disorders (see **Table 7**), neither on the overall structural level nor the structural dimensions (hypothesis 3a).

**Table 7:** Group differences in OPD-2-LSIA ratings between schizophrenia and schizoaffective disorder.

OPD-2-LSIA Dimensions	Diagnosis	N	M	SD ±	t	p
**Self-perception**	F20	96	2.61	.637	1.768	.080
	F25	33	2.39	.600		
**Object perception**	F20	96	3.05	.560	1.974	.051
	F25	33	2.82	.646		
**Self-regulation**	F20	96	2.77	.470	-.167	.868
	F25	33	2.78	.428		
**Regulation of object-relationship**	F20	96	2.79	.523	1.612	.109
	F25	33	2.63	.462		
**Internal communication**	F20	96	2.76	.601	.761	.448
	F25	33	2.67	.574		
**Communication with the external world**	F20	96	2.64	.615	1.101	.273
	F25	33	2.50	.594		
**Internal objects**	F20	96	2.97	.547	.653	.515
	F25	33	2.90	.479		
**External objects**	F20	96	3.11	.546	1.511	.133
	F25	33	2.94	.569		
**Overall structural level**	F20	96	2.84	.455	1.458	.147
	F25	33	2.70	.457		

Two-sided t-tests for independent samples: OPD-2-LSIA, Levels of Structural Integration Axis of the Operationalized Psychodynamic Diagnosis System; F20, schizophrenia; F25, schizoaffective disorder; N, number; M, mean; SD, standard deviation.

bold = significance maintained after Bonferroni-Holm method.

OPD-2-LSIA values: 1 – High level of structural integration, 1.5 – High to moderate level of structural integration, 2 – Moderate level of structural integration, 2.5 – Moderate to low level of structural integration, 3 – Low level of structural integration, 3.5 – Low to disintegrated level of structure, 4 – Disintegrated level of structure.

Regarding hypothesis 3b), no significant correlation was found between OPD-2-LSIA and factor 3 Depression, nor between OPD-2-LSIA and CDSS sum score (*r_s_* = 0.073, *p* = 0.409). Regarding the structural dimensions, after Bonferroni-Holm correction, a significant correlation was found between factor 3 Depression and Internal communication, and between CDSS and Internal communication. See **Table 3** for details.

## 4 Discussion

With the aim to explore the structural abilities of persons diagnosed with schizophrenia and schizoaffective psychosis, we first examined the participants’ structural abilities on a descriptive level. Our sample of outpatients showed an overall structural level between ‘moderate to low’ and ‘low level of structural integration’ (*M*=2.80). To the best of our knowledge there is only one pilot study (30) that previously applied OPD-LSIA to people with psychosis, which in contrast yielded a ‘low to disintegrated level of structural integration’ (*M*=3.45). However, this study consisted of a much smaller sample size (n=10) than the current study (n=129). The results of our study contradict the assumption that people with psychosis have automatically the “lowest” or “weakest” psychic structure, in the sense of a “psychotic” (76) or “disintegrated” (OPD-2-LSIA) level of structural integration. Thus, the wide range of structural abilities in our sample is noticeable (R= 1.75 - 3.90) indicating considerable heterogeneity amongst the group of people with psychosis. The greatest structural limitations were found in the structural dimensions Object perception (ability to form a realistic and wholesome picture of someone else differentiated from the self), Internal objects (including internalization, the use of introjects and variability of relationship patterns) and External objects (ability to attach, to accept help and to deal with separations and losses). Findings regarding Internal objects and External objects are in line with the results from the pilot study by Uzdawinis et al. (30), studies on the attachment style of people with psychosis (77, 78) and psychoanalytic theories that consider schizophrenia as a relationship disorder, such as the concept of an irreconcilable dilemma between self- and other-directed tendencies (53, 79, 80). Difficulties in Object perception may correspond to the well-described deficits on theory of mind and social cognition in schizophrenia (81).

Looking at previous studies on a descriptive level, it can be seen that patients with “neurotic” disorders possessed a rather ‘moderate level of structural integration’ (27, 28, 82, 83), and patients with personality disorders a ‘moderate to low level of structural integration’ (27, 28). In order to adequately compare structural abilities, a next step should be a comparative study of people with psychosis and other disorders.

### 4.1 Hypotheses 1a and 1b

Low to moderate, but significant correlations were identified between OPD-2-LSIA and the state-related indicators of severity of illness CGI-S and inpatient treatment days in the last two years and the long-term indicator lifetime hospital stays. Noteworthy is that the other long-term indicators of illness severity (duration of illness, childhood maltreatment and WHO-DDD) did not show significant correlations with OPD-2-LSIA. Our study did not confirm previous findings regarding the relationship between childhood trauma, psychic structure and psychosis. However, the role of childhood trauma was not the focus of our study and should be explored further.

Moderate associations were found between OPD-2-LSIA and the psychotic symptom factors (Factor 1 Cognition/Negative symptoms, Factor 2 Delusion/hallucinations, Factor 4 Excitement/disorganization). Participants with higher symptom load showed a lower level of structural integration. This seems plausible, since – from a psychoanalytic point of view - people with lower structural abilities are less able to cope with stressful occurrences or inner antagonisms, which can only be responded by developing psychotic symptoms and cause a need for treatment. Results of multiple linear regression analysis indicate that only acute positive, negative and disorganized psychotic symptoms, but not depressive symptoms or long-term parameters of illness severity (lifetime hospital stays, duration of illness, WHO-DDD, childhood maltreatment) predict the overall structural level. It can be assumed that the psychotic pathology itself strongly influences the person’s structural abilities: Structural abilities might be temporarily unavailable or hidden by the pathology. This corresponds to psychodynamic theories of a coexistence of psychotic and non-psychotic personality parts (84, 85): in remitted, non-florid phases, the non-psychotic personality parts take the foreground and enable psychosocial adaption. In acute, psychotic states, however, integrative functions can no longer be maintained and might decompensate - Lempa et al. (8) speak of insufficient (ego) tools for processing reality - and structural abilities are inevitably rated worse. Luyten and Fonagy (86) even argue that at higher levels of general psychopathology, e.g. in acute psychosis, it becomes impossible to distinguish between “personality and disorder”. However, results also indicate that structural abilities seem to reappear or to recover, when psychotic symptoms subside. The psychic structure of a patient could therefore also be assessed incorrectly, if depending merely on the temporary occurrence of psychotic symptoms within a 2-year assessment period. However, even if a shortened assessment period would be assumed, item descriptions such as “delusional identity aspects” (14, p. 360) or “distortions of reality perception” (14, p. 362) lead almost invariably to an assessment based on the respective psychotic pathology. This stands in contrast to the assumption of the OPD, according to which the assessment of structure is not “necessarily oriented to the current disturbance and/or its quality as an illness” (14, p. 199) and to the presumed stability of psychic structure over time. Even though OPD-2-LSIA describes a broader range of structural abilities that are only partially impacted by symptoms, the extent and frequency of possible changes in structural abilities during the assessment period are not systematically captured by the instrument. Thus, in people with disorders showing recidivism or a fluctuating course, the assessment of structural abilities might prove to be inadequate. Moreover, a volatility of structural deficits (as opposed to permanence) in people with psychosis can be seen as an inherent aspect of the disorder and might, from a clinical perspective, even indicate better prognosis.

Previous studies - conducted in patients diagnosed with other disorders - yielded inconsistent results regarding the correlation of symptom distress and psychic structure: While some authors found significant associations (26, 36, 37), others did not (33, 87). It might be assumed that the correlation presented here between pathology and structural abilities is at least partly specific to psychosis. This would also explain the lack of association between OPD-2-LSIA and depressive symptom load, which will be discussed below.

### 4.2 Hypotheses 2a and 2b

In the present sample correlations were found between OPD-2-LSIA and psychosocial functioning (Mini-ICF-APP), as well as between OPD-2-LSIA and the occupational (MVSI) and the residential status (MLCI). OPD-2-LSIA also had a relevant predictive value on the Mini-ICF-APP beyond cognition and current symptom load. This may be explained by a certain conceptual overlap. Abilities such as self- and affect regulation, to form and regulate relationships, or to anticipate other’s responses, - which are items of OPD-2-LSIA - can be understood as the underlying foundations for psychosocial functioning and participation, including social participation in the housing and the labor market. The results on the residential status are in line with the results of Spitzer et al. (33), who found better structural abilities among married people, i.e. living in a relationship, as opposed to people being single or separated. To the best of our knowledge, correlations between OPD-2-LSIA and functioning have only been investigated by Lange and Heuft (36), who used the Global Assessment of Functioning Scale (GAF) in outpatients (n=263) of a clinic for psychosomatic medicine and psychotherapy, and did not find a respective association. Despite the symptom-independent predictive power of the OPD-2-LSIA, it can be assumed that its correlation with psychosocial functioning is at least partially psychosis-specific: due to the psychotic symptom descriptions within the OPD-2-LSIA the correlation might be partially mediated by the symptomatology, whereas this is not the case for other disorders. Nevertheless, it can be assumed that an amelioration of structural abilities, e.g. through PDT, might have an impact on the psychosocial functioning of people with psychosis.

### 4.3 Hypotheses 3a and 3b

The assumption that people with schizoaffective disorders show better structural abilities than people with schizophrenia did not vindicate, as no relevant group difference was found. The findings indicate that although people with psychosis are a heterogeneous group, also in terms of psychic structure, the diagnosis and the presence of depressive (affective) symptoms are not the distinctive criteria. This is further demonstrated in the fact that no correlation between structural abilities and depressive symptoms could be found (Factor 3 Depression, CDSS; hypothesis 3b), except from the structural dimension Internal communication. The presumed ‘protective’ function of affective symptoms on structural abilities could therefore not be shown. The assumption that affective symptoms are prognostic for a good outcome in psychosis (49) is generally no longer supported in all studies (88). Moreover, depressive symptoms had no significant predictive value for OPD-2-LSIA compared to the other symptom factors. This also argues for the close association between psychosis-specific symptoms and structural abilities, as discussed above, rather than a general relationship between symptomatology and OPD-2-LSIA.

### 4.4 Limitations and outlook

Besides a pilot study by Uzdawinis et al. (30), this is the first published study on OPD-2-LSIA in schizophrenia. Due to the paucity of OPD-2-LSIA research in this patient group, interpretations of the results warrant some caution. In terms of rating the participants’ structural abilities the trained OPD-2-LSIA raters of our study reported the recurrent difficulty that item descriptions formulated in the structure checklist (14) did not depict the structural abilities of the participants unequivocally. Particularly with regard to the variability of structural abilities depending on the existence of psychotic symptoms, but also in relation to the different areas of life, item-specific structural abilities regularly exhibited parts of the ‘moderate level of structural integration’ and the ‘disintegrated level’. The checklist descriptions for ‘low level of structural integration’, however, were often not matching the structural abilities of the participants. Consequently, as compromise solutions, often interim ratings were chosen. Though, these compromise solutions do not represent an adequate solution for this problem. It must be suspected that in OPD-2-LSIA a clear distinction between symptoms and structure is hardly possible in people with psychosis. The cross-sectional design of this study does not allow for a conclusion to the question whether OPD-2-LSIA is a factor of vulnerability or an indicator of acute illness. The focus of this study is not on the possible influence of childhood trauma on the development of psychotic illness and its symptomatology: a possible mediating function of childhood trauma between structural abilities and psychotic illness must therefore be investigated further. To investigate potential relationships between specific phases of the illness (from an affective and psychotic perspective) and structural abilities, the next step would be to examine this patient group longitudinally, i.e. at different points in time, using OPD-2-LSIA. It should be noted that antipsychotic medication can interfere with mood, specifically with depressive symptoms (89). Furthermore, it should be considered that a possible influence of other pharmacological treatments (such as antidepressants or mood stabilizers) was not investigated in the study, though such influence cannot be ruled out.

Solutions could be the changes in the new OPD-3-LSIA, which was published during the implementation of the study: the lower classification levels were revised and elaborated, furthermore, ratings regarding structure variability and different life domains were proposed in the research appendix (17). These should be comparatively validated on different clinical samples and should also address the question regarding the relationship of structure and symptom load.

### 4.5 Conclusion

In conclusion, the psychic structure of people with psychosis is more heterogeneous than usually assumed. Associations between psychic structure and psychosocial functioning as well as (psychotic) symptomatology were shown. The results seem to argue against the thesis of a rather invariant psychic structure and for more changeable structural abilities. They suggest that there are structural deficits in the patient group, which worsen with the increase in state-related factors of the illness such as acute psychotic symptoms. In contrast, rather trait-related factors seem to have less influence. However, this model needs to be tested in a longitudinal study. Also, no significant differences were found between schizophrenia and schizoaffective disorders.

The results of our study regarding the impairments of psychic structure and its subdimensions illustrate that a detailed examination of “personality functioning” in relation to the self and others can be useful for patients with psychotic disorders - and not only for personality disorders as conceptualized in the AMPD (16). Despite the limitations described above the results also show the applicability of the OPD-2-LSIA in people with psychosis. Especially with the upcoming changes in the new OPD-3-LSIA it might become an important tool in research and psychotherapy planning for people with psychosis.

## Data availability statement

The raw data supporting the conclusions of this article will be made available by the authors, without undue reservation.

## Ethics statement

The studies involving humans were approved by Ethics committee of the Charité Universitätsmedizin Berlin. The studies were conducted in accordance with the local legislation and institutional requirements. The participants provided their written informed consent to participate in this study.

## Author contributions

SB: Writing – original draft, Writing – review & editing. A-LB: Writing – review & editing. FS: Writing – review & editing. SJ: Writing – review & editing. GB: Writing – review & editing. IG: Writing – review & editing. EM: Writing – review & editing. MB: Writing – review & editing. AH: Writing – review & editing. FB: Writing – review & editing. GL: Writing – review & editing. DH: Writing – review & editing. CM: Writing – review & editing.

## References

[1] Gaebel W Hasan A Falkai P. S3-leitlinie schizophrenie. Berlin: Springer-Verlag (2019). doi: 10.1007/978-3-662-59380-6

[2] National Collaborating Centre for Mental Health (NCCMH). Psychosis and schizophrenia in adults. In: The nice guidelines on treatment and management. National Institute for Health and Care Excellence, London (2014).

[3] Lincoln TM Pedersen A. An overview of the evidence for psychological interventions for psychosis: Results from meta-analyses. Clin Psychol Europe. (2019) 1:1–23. doi: 10.32872/cpe.v1i1.31407

[4] Sitko K Bewick BM Owens D Masterson C. Meta-analysis and meta-regression of cognitive behavioral therapy for psychosis (CBTp) across time: the effectiveness of CBTp has improved for delusions. Schizophr Bull Open. (2020) 1. doi: 10.1093/schizbullopen/sgaa023

[5] Turner DT van der Gaag M Karyotaki E Cuijpers P. Psychological interventions for psychosis: A meta-analysis of comparative outcome studies. Am J Psychiatry. (2014) 171:523–38. doi: 10.1176/appi.ajp.2013.13081159 24525715

[6] Malmberg L Fenton M. Individual psychodynamic psychotherapy and psychoanalysis for schizophrenia and severe mental illness. Cochrane Database Systematic Rev. (2001) 1–42. doi: 10.1002/14651858.CD001360 PMC4171459 11686988

[7] Weijers J Ten Kate C Viechtbauer W Rampaart L Eurelings E Selten J. Mentalization-based treatment for psychotic disorder: A rater-blinded, multi-center, randomized controlled trial. psychol Med. (2021) 51:2846–55. doi: 10.1017/S0033291720001506 PMC8640364 32466811

[8] Lempa G von Haebler D Montag C. Psychodynamische psychotherapie der schizophrenien. Ein manual. 2th Edn. Gießen: Psychosozial-Verlag (2017). doi: 10.30820/9783837972214

[9] Rosenbaum B Harder S Knudsen P Køster A Lindhardt A Lajer M. Supportive psychodynamic psychotherapy versus treatment as usual for first-episode psychosis: two-year outcome. Psychiatry: Interpersonal Biol Processes. (2012) 75:331–41. doi: 10.1521/psyc.2012.75.4.331 23244011

[10] Grande T Rudolf G Oberbracht C. Die Strukturachse der Operationalisierten Psychodynamischen Diagnostik (OPD): Forschungsergebnisse zum Konzept und zur klinischen Anwendung. [The structure axis Operationalize Psychodynamic Diagnosis (OPD): Res findings its concept Clin application]. Persönlichkeitsstörungen - Theorie Und Therapie. (1998) . 4:173–82.

[11] Henkel M Zimmermann J Huber D Staats H Wiegand-Grefe S Taubner S. Patient characteristics in psychodynamic psychotherapies. Psychoanalytic Psychol. (2019) 36:1–8. doi: 10.1037/pap0000165

[12] Rudolf G Buchheim P Ehlers W Küchenhoff J Muhs A Pouget-Schors D. Struktur und strukturelle Störung. Z für Psychosomatische Med und Psychoanalyse. (1995) 41:197–212. Available online at: http://www.jstor.org/stable/23997695. 7571881

[13] Abraham K. Psychoanalytische studien zur charakterbildung. Internationale Psychoanalytische Bibliothek. (1925) 16:1–64.

[14] Task Force OPD. Operationalized Psychodynamic Diagnosis OPD-2: Manual of diagnosis and treatment planning, 1st edition. Kirkland: Hogrefe. (2008) 1–407.

[15] Kernberg O Levy KN. Borderline-Persönlichkeitsstörung und Borderline-Persönlichkeitsorganisation – Psychopathologie und Diagnose. In: Kernberg O Dulz B Sachsse U, editors. Handbuch der borderline-störungen. Schattauer, Stuttgart (2000). p. 286–301.

[16] American Psychiatric Association. Diagnostic and statistical manual of mental disorders (DSM-5 (R)). 5th Edn. Washington, DC: American Psychiatric Association (2013). doi: 10.1176/appi.books.9780890425596

[17] Arbeitskreis OPD. Operationalisierte psychodynamische diagnostik OPD-3. Das Manual für Diagnostik und Therapieplanung. Bern: Huber (2023). doi: 10.1024/86263-000

[18] Zimmermann J Ehrenthal JC Cierpka M Schauenburg H Doering S Benecke C. Assessing the level of structural integration using operationalized psychodynamic diagnosis (OPD): implications for DSM-5. J Pers Assess. (2012) 94:522–32. doi: 10.1080/00223891.2012.700664 22808938

[19] Zimmermann J. Assessing the DSM-5 Level of Personality Functioning and the OPD Level of Structural Integration amounts to the same thing. Eur Soc Study Pers Disord (ESSPD) Newslett. (2014) 3:9–10.

[20] Doering S Blüml V Parth K Feichtinger K Gruber M Aigner M. Personality functioning in anxiety disorders. BMC Psychiatry. (2018) 18:1–9. doi: 10.1186/s12888-018-1870-0 30223818 PMC6142416

[21] Clarkin JF Caligor E Stern B Kernberg O. Structured interview of personality organization (STIPO). New York: Weill Medical College of Cornell University (2004).

[22] DeWitt KN Hartley DE Rosenberg SE Zilberg NJ Wallerstein RS. Scales of psychological capacities: Development of an assessment approach. Psychoanalysis Contemp Thought. (1991) 14:343–61.

[23] Doering S Burgmer M Heuft G Menke D Bäumer B Lübking M. Assessment of personality functioning: Validity of the Operationalized Psychodynamic Diagnosis Axis IV (structure). Psychopathology. (2013) 47:185–93. doi: 10.1159/000355062 24192300

[24] Ehrenthal JC. Strukturdiagnostik. Neue Ergebnisse aus der Forschung für die Praxis. [Measuring structural integration. New results from research for practical settings]. Psychodynamische Psychotherapie. (2014) 13:103–14.

[25] Rudolf G Doering S. “Die Strukturachse der Operationalisierten Psychodynamischen Diagnostik (OPD-2)”. In: Doering S Hörz S, editors. Handbuch der Strukturdiagnostik: Konzepte, Instrumente, Praxis. Stuttgart: Schattauer (2012). p. 87–120.

[26] Benecke C Koschier A Peham D Bock A Dahlbender RW Biebl W. Erste Ergebnisse zu Reliabilität und Validität der OPD-2 Strukturachse. [First results on the reliability and validity of the OPD-2 axis structure]. Z für Psychosomatische Med und Psychotherapie. (2009) 55:84–102. doi: 10.13109/zptm.2009.55.1.84 19353514

[27] Menke BKM. Die Operationalisierte Psychodynamische Diagnostik (OPD-2): Strukturniveau und psychiatrische Diagnose. Münster (Germany: Medizinische Fakultaüt der Westfaülischen Willhelms-Universitaüt Münster (2011).

[28] Nitzgen D Brünger M. “Operationalisierte Psychodynamische Diagnostik in der Rehabilitationsklinik Birkenbruck: Einsatz und Befunde”. In: Schneider W Freyberger HJ, editors. Was leistet die OPD? Empirische Befunde und klinische Erfahrungen mit der Operationalisierten Psychodynamischen Diagnostik. Bern: Huber (2000). p. 238–52.

[29] Rudolf G Jakobsen T Grande T Oberbracht C. “Strukturelle Aspekte der Persönlichkeitsstörungen”. In: Rudolf G Grande T Henningsen P, editors. Die Struktur der Persönlichkeit. Vom theoretischen Verständnis zur therapeutischen Anwendung des psychodynamischen Strukturkonzepts. Schattauer, Stuttgart (2002). p. 158–76.

[30] Uzdawinis D Edel M Özgürdal S von Haebler D Hauser M Witthaus H. Operationalisierte Psychodynamische Diagnostik (OPD) bei Patienten im schizophrenen Prodromalstadium - Eine explorative Studie. [Operationalized psychodynamic diagnostics (OPD) in patients in a prodromal state of schizophrenia - An explorative study]. Z für Psychosomatische Med und Psychotherapie. (2010) 56:150–62. doi: 10.13109/zptm.2010.56.2.150 20623460

[31] Himmighoffen H Böker H. Operationalisierte Psychodynamische Diagnostik (OPD) in der Psychotherapie von Patienten mit bipolarer affektiver Störung [Operationalized Psychodynamic Diagnosis (OPD) in the Psychotherapy of Patients with Bipolar Affective Disorder]. Psychotherapie In Psychiatrie Psychotherapeutischer Med und Klinischer Psychol. (2011) 16:56–64.

[32] Rapaport D. The structure of psychoanalytic theory. psychol Issues. (1960) 2:1–158.

[33] Spitzer C Michels-Lucht F Siebel U Freyberger H. Die Strukturachse der operationalisierten psychodynamischen Diagnostik (OPD): Zusammenhänge mit soziodemographischen, klinischen und psychopathologischen Merkmalen sowie kategorialen Diagnosen. [The Axis „Structure” of the Operationalized Psychodynamic Diagnostics (OPD): Its Relationship with Sociodemographic, Clinical and Psychopathological Features as Well as Categorical Diagnoses]. Psychotherapie Psychosomatik Medizinische Psychol. (2002) 52:392–97. doi: 10.1055/s-2002-34290 12355346

[34] Thomasius R Weiler D Sack PM Schindler A Gemeinhardt B Schuhbert C. Validität der Operationalisierten Psychodynamischen Diagnostik (OPD) bei familientherapeutisch behandelten Drogenabhängigen im adoleszenten und jungen Erwachsenenalter. [Validity of Operationalized Psychodynamic Diagnostics (OPD) in the Field of Family Therapy with Adolescent and Young Adult Drug Addicts]. PPmP-Psychotherapie Psychosomatik Medizinische Psychol. (2001) 51:365–72. doi: 10.1055/s-2001-16896 11533883

[35] Arbeitskreis OPD. Operationalisierte psychodynamische diagnostik OPD. Das Manual für Diagnostik und Therapieplanung. Grundlagen und Manual. Bern: Huber (2001).

[36] Lange C Heuft G. Die Beeinträchtigungsschwere in der psychosomatischen und psychiatrischen Qualitätssicherung: Global Assessment of Functioning Scale (GAF) vs. Beeinträchtigungs-Schwere-Score (BSS). [Psychic impairment in psychosomatic and psychiatric quality assessment: global assessment of functioning scale (GAF) vs. impairment Score (IS)]. Z Für Psychosomatische Med und Psychotherapie. (2002) 48:256–69. doi: 10.13109/zptm.2002.48.3.256 12136447

[37] Mestel R Klingelhöfer J Dahlbender R Schüßler G. “Validität der OPD-Achsen. Konflikt und Struktur in der stationären psychosomatischen Rehabilitation”. In: Dahlbender R Buchheim P Schüßler G, editors. Lernen an der Praxis. OPD und Qualitätssicherung in der Psychodynamischen Psychotherapie. Bern: Huber (2004). p. 229–44.

[38] Ehrenthal JC Dinger U Schauenburg H Horsch L Dahlbender RW Gierk B. Entwicklung einer Zwölf-Item-Version des OPD-Strukturfragebogens (OPD-SFK). [Development of a 12-item version of the OPD-Structure Questionnaire (OPD-SQS)]. Z für Psychosomatische Med und Psychotherapie. (2015) 61:262–74. doi: 10.13109/zptm.2015.61.3.262 26388057

[39] Baie L Hucklenbroich K Hampel N Ehrenthal JC Heuft G Burgmer M. Steht das strukturelle Integrationsniveau nach OPD-2 in Zusammenhang mit der Symptomschwere einer Posttraumatischen Belastungsstörung (PTBS)?–Eine Kohortenstudie bei Patienten einer Trauma-Ambulanz. [Level of personality functioning (OPD-2) and the symptom severity of posttraumatic stress dis- order – a cohort study]. Z für Psychosomatische Med und Psychotherapie. (2020) 66:5–19. doi: 10.13109/zptm.2020.66.1.5 32066355

[40] Wagner-Skacel J Bengesser S Dalkner N Mörkl S Painold A Hamm C. Personality structure and attachment in bipolar disorder. Front Psychiatry. (2020) 11:410. doi: 10.3389/fpsyt.2020.00410 32477186 PMC7233168

[41] Hintermeier S. Traumatisierung im Kindesalter und strukturelle Störungen. Z Psychodrama Soziom. (2021) 20:9–22. doi: 10.1007/s11620-021-00623-y

[42] Varchmin L Montag C Treusch Y Kaminski J Heinz A. Traumatic events, social adversity and discrimination as risk factors for psychosis - an umbrella review. Front Psychiatry. (2021) 12:665957. doi: 10.3389/fpsyt.2021.665957 34744806 PMC8569921

[43] Brissos S Molodynski A Dias VV Figueira ML. The importance of measuring psychosocial functioning in schizophrenia. Ann Gen Psychiatry. (2011) 10. doi: 10.1186/1744-859X-10-18 PMC3132204 21702932

[44] Benabarre A Vieta E Colom F Martínez-Arán A Reinares M Gastó C. Bipolar disorder, schizoaffective disorder and schizophrenia: Epidemiologic, clinical and prognostic differences. Eur Psychiatry. (2001) 16:167–72. doi: 10.1016/S0924-9338(01)00559-4 11353595

[45] Marneros A Deister A Rohde A. Comparison of long-term outcome of schizophrenic, affective and schizoaffective disorders. Br J Psychiatry. (1992) 161:44–51. doi: 10.1192/S0007125000297043 1389041

[46] Bock A Huber E Müller S Henkel M Sevecke K Schopper A. Psychisches Strukturniveau im Jugendalter und der Zusammenhang mit späterer psychischer Erkrankung – eine Langzeitstudie. [Levels of structural integration in adolescents and the relationship to later mental disorders – A longitudinal study]. Z für Kinder-und Jugendpsychiatrie und Psychotherapie. (2019) 47:400–10. doi: 10.1024/1422-4917/a000656 30939974

[47] Kraepelin E. Psychiatrie: Ein Lehrbuch für Studierende und Ärzte. In: [Psychiatry: a manual for students and physicians]. Barth, Leipzig (1913).

[48] Taylor MA Abrams R. Manic-depressive illness and good prognosis schizophrenia. Am J Psychiatry. (1975) 132:741–42. doi: 10.1176/ajp.132.7.741 1137021

[49] Pope HG Jr Lipinski JF Jr. Diagnosis in schizophrenia and manic-depressive illness: a reassessment of the specificity of 'schizophrenic' symptoms in the light of current research. Arch Gen Psychiatry. (1978) 35:811–28. doi: 10.1001/archpsyc.1978.01770310017001 354552

[50] Bröcker AL Bayer S Stuke F Just S Bertram G Funcke J. Levels of structural integration mediate the impact of metacognition on functioning in non-affective psychosis: Adding a psychodynamic perspective to the metacognitive approach. Front Psychol. (2020) 11:269. doi: 10.3389/fpsyg.2020.00269 32153475 PMC7047329

[51] Just SA Haegert E Kořánová N Bröcker AL Nenchev I Funcke J. Modeling incoherent discourse in non-affective psychosis. Front Psychiatry. (2020) 11:846. doi: 10.3389/fpsyt.2020.00846 32973586 PMC7466436

[52] Maaßen E Büttner M Bröcker AL Stuke F Bayer S Hadzibegovic J. Measuring emotional awareness in patients with schizophrenia and schizoaffective disorders. Front Psychol. (2021) 12:725787. doi: 10.3389/fpsyg.2021.725787 34858263 PMC8632138

[53] Stuke F Bröcker AL Bayer S Heinz A Bermpohl F Lempa G. Between a rock and a hard place: Associations between Mentzos' “dilemma”, self-reported interpersonal problems, and psychosocial functioning in individuals with non-affective psychoses. Clin Psychol Psychother. (2020) 27:528–41. doi: 10.1002/cpp.2437 32100357

[54] American Psychiatric Association. Diagnostic and statistical manual of mental disorders. 4th Edn. Washington, DC: American Psychiatric Association (2000).

[55] Wittchen H-U Wunderlich U Gruschwitz S Zaudig M. SKID-I. Strukturiertes klinisches interview für DSM-IV. Achse I: psychische störungen [SCID-I. Structured clinical interview for DSM-IV. Axis I disorders]. Göttingen: Hogrefe AG (1997).

[56] World Health Organization. Collaborating centre for drug statistics methodology (2018). ATC/DDD index (2019). Available online at: https://www.whocc.no/atc_ddd_index/ (Accessed September 27, 2023).

[57] Kay SR Fiszbein A Opler LA. The positive and negative syndrome scale (PANSS) for schizophrenia. Schizophr Bull. (1987) 13:261–76. doi: 10.1093/schbul/13.2.261 3616518

[58] Citrome L Meng X Hochfeld M. Efficacy of iloperidone in schizophrenia: A PANSS five-factor analysis. Schizophr Res. (2011) 131:75–81. doi: 10.1016/j.schres.2011.05.018 21700430

[59] Andreasen NC. Scale for the assessment of positive symptoms (SAPS). Iowa City: University of Iowa (1984).

[60] Andreasen NC. The scale for the assessment of negative symptoms (SANS). Iowa City: University of Iowa (1983).

[61] Müller MJ Marx-Dannigkeit P Schlösser R Wetzel H Addington D Benkert O. The Calgary Depression Rating Scale for Schizophrenia: development and interrater reliability of a German version (CDSS-G). J Psychiatr Res. (1999) 33:433–43. doi: 10.1016/S0022-3956(99)00018-7 10504012

[62] Kay SR Opler LA Lindenmayer JP. Reliability and validity of the positive and negative syndrome scale for schizophrenics. Psychiatry Res. (1988) 23:99–110. doi: 10.1016/0165-1781(88)90038-8 3363019

[63] Müller MJ Rossbach W Davids E Wetzel H Benkert O. Evaluation eines standardisierten Trainings für die “Positive and Negative Syndrome Scale” (PANSS). Der Nervenarzt. (2000) 71:195–204. doi: 10.1007/s001150050029 10756528

[64] Strauß B Schumacher J. Klinische interviews und ratingskalen. Göttingen: Hogrefe Verlag (2004).

[65] Guy W. Clinical global impressions (CGI) scale. ECDEU Assessment Manual for Psychopharmacology. Rockville, MD: U.S. Department of Health,Education, and Welfare (1976).

[66] Grabe H Schulz A Schmidt C Appel K Driessen M Wingenfeld K. Ein Screeninginstrument für Missbrauch und Vernachlässigung in der Kindheit: der Childhood Trauma Screener (CTS) [A brief instrument for the assessment of childhood abuse and neglect: the childhood trauma screener (CTS). Psychiat Prax. (2012) 39:109–15. doi: 10.1055/s-0031-1298984 22422160

[67] Linden M Baron S Muschalla B. “Mini-ICF-Rating für psychische Stoürungen (Mini-ICF-APP)”. In: Ein Kurzinstrument zur Beurteilung von Fähigkeits- bzw. Kapazitätsstörungen bei psychischen Störungen. Göttingen: Hans Huber (2009).

[68] World Health Organization. International classification of functioning, disability and health: ICF. Geneva, Switzerland: World Health Organization (2001).

[69] Molodynski A Linden M Juckel G Yeeles K Anderson C Vazquez-Montes M. The reliability, validity, and applicability of an English language version of the Mini-ICF-APP. Soc Psychiatry Psychiatr Epidemiol. (2013) 48:1347–54. doi: 10.1007/s00127-012-0604-8 23080483

[70] Baron S. Operationalisierung und Quantifizierung von Fähigkeitsstörungen bei psychischen Erkrankungen. Berlin (Germany: Freie Universität Berlin (2011).

[71] Tohen M Waternaux CM Tsuang MT. Outcome in mania: A 4-year prospective follow-up of 75 patients utilizing survival analysis. Arch Gen Psychiatry. (1990) 47:1106–11. doi: 10.1001/archpsyc.1990.01810240026005 2244795

[72] Heubrock D. Der Auditiv-Verbale Lerntest (AVLT) in der klinischen und experimentellen Neuropsychologie. Durchführung, Auswertung und Forschungsergebnisse [The Auditory-Verbal Learning Test (AVLT) in clinical and experimental neuropsychology: Administration, evaluation, and research findings]. Z für Differentielle und Diagnostische Psychol. (1992) 13:161–74.

[73] Schmidt K-H Metzler P. Wortschatztest (WST). Beltz Test GmbH: Weinheim, 1st edition. (1992).

[74] IBM Corp. IBM SPSS statistics for macintosh, version 23.0. Armonk, NY: IBM Corp (2015).

[75] Akaike H. Information theory as an extension of the maximum likelihood principle. In: Petrov BN Csaki F, editors. Second international symposium on information theory. Akademiai Kiado, Budapest (1973). p. 267–81.

[76] Kernberg O. Psychotic personality structure. Psychodynamic Psychiatry. (2019) 47:353–72. doi: 10.1521/pdps.2019.47.4.353 31913791

[77] Berry K Barrowclough C Wearden A. Attachment theory: a framework for understanding symptoms and interpersonal relationships in psychosis. Behav Res Ther. (2008) 46:1275–82. doi: 10.1016/j.brat.2008.08.009 18926521

[78] Gumley AI Taylor HEF Schwannauer M MacBeth A. A systematic review of attachment and psychosis: measurement, construct validity and outcomes. Acta Psychiatrica Scandinavica. (2014) 129:257–74. doi: 10.1111/acps.12172 23834647

[79] Lempa G Böker H. Theorie und Therapie der schizophrenen Psychose aus psychoanalytischer Sicht. Psychotherapie. (1999) 4:98–106.

[80] Mentzos S. Lehrbuch der Psychodynamik: Die Funktion der Dysfunktionalität psychischer Störungen. Göttingen: Vandenhoeck & Ruprecht (2009).

[81] Savla GN Vella L Armstrong CC Penn DL Twamley EW. Deficits in domains of social cognition in schizophrenia: a meta-analysis of the empirical evidence. Schizophr Bull. (2013) 39:979–92. doi: 10.1093/schbul/sbs080 PMC3756768 22949733

[82] Böker H Himmighoffen H Straub M Schopper C Endrass J Kuechenhoff B. Deliberate self-harm in female patients with affective disorders: Investigation of personality structure and affect regulation by means of operationalized psychodynamic diagnostics. J Nervous Ment Dis. (2008) 196:743–51. doi: 10.1097/NMD.0b013e3181879daf 18852618

[83] Kaufhold J Negele A Leuzinger-Bohleber M Kallenbach L Ernst M Bahrke U. Zur Konfliktdynamik bei chronischer Depression - Ergebnisse zur Konflikt-und Strukturachse der OPD in der LAC-Studie. [Conflict dynamics in chronic depression - Results of the conflict and structure axis using the OPD in the LAC Study]. Z für Psychosomatische Med und Psychotherapie. (2017) 63:151–62. doi: 10.13109/zptm.2017.63.2.151 28585509

[84] Bion WR. Zur Unterscheidung von psychotischen und nicht-psychotischen Persönlichkeiten [Differentiation of the psychotic from the non-psychotic personalities]. In: Bott Spillius E, editor. Melanie Klein Heute. Entwicklungen in Theorie und Praxis, Band 1 Beiträge zur Theorie. Klett-Cotta, Stuttgart (2002). p. 75–102.

[85] Müller T. Über psychotische persönlichkeitsorganisationen. In: Schwarz F Maier C, editors. Psychotherapie der psychosen. Thieme, Stuttgart (2001). p. 28–37.

[86] Luyten P Fonagy P. Integrating and differentiating personality and psychopathology: A psychodynamic perspective. J Pers. (2022) 90:75–88. doi: 10.1111/jopy.12656 34170512

[87] Spitzer C Michels-Lucht F Siebel U Freyberger H. Zum Zusammenhang zwischen OPD-Merkmalen der Persönlichkeitsstruktur und symptombezogenen sowie interpersonalen Behandlungsergebnissen stationärer Psychotherapie. [On the relationship between OPD features of personality structure and symptom-related and interpersonal outcome of inpatient psychotherapy]. Z Für Psychosomatische Med und Psychotherapie. (2004) 50:70–85. doi: 10.13109/zptm.2004.50.1.70 14747984

[88] Grossman LS Harrow M Goldberg JF Fichtner CG. Outcome of schizoaffective disorder at two long-term follow-ups: comparisons with outcome of schizophrenia and affective disorders. Am J Psychiatry. (1991) 148:1359–65. doi: 10.1176/ajp.148.10.1359 1897617

[89] Reynolds GP McGowan OO. Schizophrenia, depressive symptoms, and antipsychotic drug treatment. Int J Neuropsychopharmacol. (2021) 24:253–55. doi: 10.1093/ijnp/pyaa091 PMC8059489 33882123

