# Supplementary material for: Level of structural integration in people with schizophrenia and schizoaffective disorders - applicability and associations with clinical parameters
Source: Front Psychiatry. 2024 Jun 6;15:1388478. doi: 10.3389/fpsyt.2024.1388478 (PMC11192590; doi:10.3389/fpsyt.2024.1388478)
Supplement: Supplementary file 1 [file Table_1.docx]

**Level of Structural Integration in people with schizophrenia and schizoaffective disorders - applicability and associations with clinical parameters**

**Supplements:**

Modified Vocational Status Index (MVSI)

1, employed full-time at expected level (100% or at least 50% if having the additional status of an active student); 2, employed full-time below expected level; 3, employed part-time; 4, vocational (re-)training including vocational rehabilitation or trainings as a peer-support worker; 5, retired with a small additional income, doing volunteer work or being an active housewife / -husband; 6, employed in a sheltered workshop; 7, occupational therapy, regular activity in a day care center for subjects with mental disorders; 8, long-term unemployed but employable and 9, retired or unable to work.

Modified Location Code Index (MLCI)

1, living as a parent with children, in a partnership, with friends or relatives (criterium: living in a relationship); 2, in an autonomous household alone, with peers or a shared apartment (criterium: living without a relevant relationship); 3, with the nuclear family; 4, in an autonomous household with minimal supervision (e.g. integrated care, professional family assistance); 5, in a therapeutic residential community; 6, in a household with intensive supervision (e.g. assisted living, daily nursing service); 7, in transitional housing with intensive care; 8, a nursing home; 9, a permanent homeless shelter and 10, homeless, on the streets.
